# Supplementary material for: HIVprotI: an integrated web based platform for prediction and design of HIV proteins inhibitors
Source: J Cheminform. 2018 Mar 9;10:12. doi: 10.1186/s13321-018-0266-y (PMC5845081; doi:10.1186/s13321-018-0266-y)
Supplement: Supplementary file 2 — Additional file 2. Source code of HIVProtI web server. [file 13321_2018_266_MOESM2_ESM.zip › HIVprotI_Source-code/hivprotI/weka-3-6-6/doc/weka/clusterers/MakeDensityBasedClusterer.html]

MakeDensityBasedClusterer


---


|  |  |  |
| --- | --- | --- |
| **Overview** \| **Package** \| **Class** \| **Tree** \| **Deprecated** \| **Index** \| **Help** \| **Weka's home** | |  |
| **PREV CLASS**   **NEXT CLASS** | **FRAMES**    **NO FRAMES**     **All Classes** |  |
| SUMMARY: NESTED \| FIELD \| CONSTR \| METHOD | DETAIL: FIELD \| CONSTR \| METHOD |  |


---


## weka.clusterers Class MakeDensityBasedClusterer

```
java.lang.Object
  weka.clusterers.AbstractClusterer
      weka.clusterers.AbstractDensityBasedClusterer
          weka.clusterers.MakeDensityBasedClusterer
```

**All Implemented Interfaces:**: java.io.Serializable, java.lang.Cloneable, Clusterer, DensityBasedClusterer, NumberOfClustersRequestable, CapabilitiesHandler, OptionHandler, RevisionHandler, WeightedInstancesHandler

---

``` public class MakeDensityBasedClusterer extends AbstractDensityBasedClusterer implements NumberOfClustersRequestable, OptionHandler, WeightedInstancesHandler ```

Class for wrapping a Clusterer to make it return a distribution and density. Fits normal distributions and discrete distributions within each cluster produced by the wrapped clusterer. Supports the NumberOfClustersRequestable interface only if the wrapped Clusterer does.


Valid options are:

```
 -M <num>
  minimum allowable standard deviation for normal density computation 
  (default 1e-6)
```

```
 -W <clusterer name>
  Clusterer to wrap.
  (default weka.clusterers.SimpleKMeans)
```

```
 Options specific to clusterer weka.clusterers.SimpleKMeans:
```

```
 -N <num>
  number of clusters.
  (default 2).
```

```
 -V
  Display std. deviations for centroids.
```

```
 -M
  Replace missing values with mean/mode.
```

```
 -S <num>
  Random number seed.
  (default 10)
```

Options after "--" are passed on to the base clusterer.

**Version:**
:   $Revision: 5538 $

**Author:**
:   Richard Kirkby (rkirkby@cs.waikato.ac.nz), Mark Hall (mhall@cs.waikato.ac.nz), Eibe Frank (eibe@cs.waikato.ac.nz)

**See Also:**: Serialized Form

---

**Constructor Summary**

`MakeDensityBasedClusterer()`
  
          Default constructor.

`MakeDensityBasedClusterer(Clusterer toWrap)`
  
          Contructs a MakeDensityBasedClusterer wrapping a given Clusterer.


| **Method Summary** | |
| --- | --- |
| `void` | `buildClusterer(Instances data)`             Builds a clusterer for a set of instances. |
| `java.lang.String` | `clustererTipText()`             Returns the tip text for this property |
| `double[]` | `clusterPriors()`             Returns the cluster priors. |
| `Capabilities` | `getCapabilities()`             Returns default capabilities of the clusterer (i.e., of the wrapper clusterer). |
| `Clusterer` | `getClusterer()`             Gets the clusterer being wrapped. |
| `double` | `getMinStdDev()`             Get the minimum allowable standard deviation. |
| `java.lang.String[]` | `getOptions()`             Gets the current settings of the clusterer. |
| `java.lang.String` | `getRevision()`             Returns the revision string. |
| `java.lang.String` | `globalInfo()`             Returns a string describing classifier |
| `java.util.Enumeration` | `listOptions()`             Returns an enumeration describing the available options.. |
| `double[]` | `logDensityPerClusterForInstance(Instance inst)`             Computes the log of the conditional density (per cluster) for a given instance. |
| `static void` | `main(java.lang.String[] argv)`             Main method for testing this class. |
| `java.lang.String` | `minStdDevTipText()`             Returns the tip text for this property |
| `int` | `numberOfClusters()`             Returns the number of clusters. |
| `void` | `setClusterer(Clusterer toWrap)`             Sets the clusterer to wrap. |
| `void` | `setMinStdDev(double m)`             Set the minimum value for standard deviation when calculating normal density. |
| `void` | `setNumClusters(int n)`             Set the number of clusters to generate. |
| `void` | `setOptions(java.lang.String[] options)`             Parses a given list of options. |
| `java.lang.String` | `toString()`             Returns a description of the clusterer. |

**Methods inherited from class weka.clusterers.AbstractDensityBasedClusterer**

`distributionForInstance, logDensityForInstance, logJointDensitiesForInstance, makeCopies`

**Methods inherited from class weka.clusterers.AbstractClusterer**

`clusterInstance, forName, makeCopies, makeCopy`

**Methods inherited from class java.lang.Object**

`equals, getClass, hashCode, notify, notifyAll, wait, wait, wait`

**Methods inherited from interface weka.clusterers.Clusterer**

`clusterInstance`

**Constructor Detail**

### MakeDensityBasedClusterer

```
public MakeDensityBasedClusterer()
```

:   Default constructor.

---


### MakeDensityBasedClusterer

```
public MakeDensityBasedClusterer(Clusterer toWrap)
```

:   Contructs a MakeDensityBasedClusterer wrapping a given Clusterer.

    **Parameters:**: `toWrap` - the clusterer to wrap around


**Method Detail**

### globalInfo

```
public java.lang.String globalInfo()
```

:   Returns a string describing classifier

    :   **Returns:**: a description suitable for displaying in the explorer/experimenter gui

---


### setNumClusters

```
public void setNumClusters(int n)
                    throws java.lang.Exception
```

:   Set the number of clusters to generate.

    :   **Specified by:**: `setNumClusters` in interface `NumberOfClustersRequestable`
    :   **Parameters:**: `n` - the number of clusters to generate **Throws:**: `java.lang.Exception` - if the wrapped clusterer has not been set, or if the wrapped clusterer does not implement this facility.

---


### getCapabilities

```
public Capabilities getCapabilities()
```

:   Returns default capabilities of the clusterer (i.e., of the wrapper
    clusterer).

    :   **Specified by:**: `getCapabilities` in interface `Clusterer` **Specified by:**: `getCapabilities` in interface `CapabilitiesHandler` **Overrides:**: `getCapabilities` in class `AbstractClusterer`
    :   **Returns:**: the capabilities of this clusterer **See Also:**: `Capabilities`

---


### buildClusterer

```
public void buildClusterer(Instances data)
                    throws java.lang.Exception
```

:   Builds a clusterer for a set of instances.

    :   **Specified by:**: `buildClusterer` in interface `Clusterer` **Specified by:**: `buildClusterer` in class `AbstractClusterer`
    :   **Parameters:**: `data` - the instances to train the clusterer with **Throws:**: `java.lang.Exception` - if the clusterer hasn't been set or something goes wrong

---


### clusterPriors

```
public double[] clusterPriors()
```

:   Returns the cluster priors.

    :   **Specified by:**: `clusterPriors` in interface `DensityBasedClusterer` **Specified by:**: `clusterPriors` in class `AbstractDensityBasedClusterer`
    :   **Returns:**: the cluster priors

---


### logDensityPerClusterForInstance

```
public double[] logDensityPerClusterForInstance(Instance inst)
                                         throws java.lang.Exception
```

:   Computes the log of the conditional density (per cluster) for a given instance.

    :   **Specified by:**: `logDensityPerClusterForInstance` in interface `DensityBasedClusterer` **Specified by:**: `logDensityPerClusterForInstance` in class `AbstractDensityBasedClusterer`
    :   **Parameters:**: `inst` - the instance to compute the density for **Returns:**: an array containing the estimated densities **Throws:**: `java.lang.Exception` - if the density could not be computed successfully

---


### numberOfClusters

```
public int numberOfClusters()
                     throws java.lang.Exception
```

:   Returns the number of clusters.

    :   **Specified by:**: `numberOfClusters` in interface `Clusterer` **Specified by:**: `numberOfClusters` in class `AbstractClusterer`
    :   **Returns:**: the number of clusters generated for a training dataset. **Throws:**: `java.lang.Exception` - if number of clusters could not be returned successfully

---


### toString

```
public java.lang.String toString()
```

:   Returns a description of the clusterer.

    :   **Overrides:**: `toString` in class `java.lang.Object`
    :   **Returns:**: a string containing a description of the clusterer

---


### clustererTipText

```
public java.lang.String clustererTipText()
```

:   Returns the tip text for this property

    :   **Returns:**: tip text for this property suitable for displaying in the explorer/experimenter gui

---


### setClusterer

```
public void setClusterer(Clusterer toWrap)
```

:   Sets the clusterer to wrap.

    :   **Parameters:**: `toWrap` - the clusterer

---


### getClusterer

```
public Clusterer getClusterer()
```

:   Gets the clusterer being wrapped.

    :   **Returns:**: the clusterer

---


### minStdDevTipText

```
public java.lang.String minStdDevTipText()
```

:   Returns the tip text for this property

    :   **Returns:**: tip text for this property suitable for displaying in the explorer/experimenter gui

---


### setMinStdDev

```
public void setMinStdDev(double m)
```

:   Set the minimum value for standard deviation when calculating
    normal density. Reducing this value can help prevent arithmetic
    overflow resulting from multiplying large densities (arising from small
    standard deviations) when there are many singleton or near singleton
    values.

    :   **Parameters:**: `m` - minimum value for standard deviation

---


### getMinStdDev

```
public double getMinStdDev()
```

:   Get the minimum allowable standard deviation.

    :   **Returns:**: the minumum allowable standard deviation

---


### listOptions

```
public java.util.Enumeration listOptions()
```

:   Returns an enumeration describing the available options..

    :   **Specified by:**: `listOptions` in interface `OptionHandler`
    :   **Returns:**: an enumeration of all the available options.

---


### setOptions

```
public void setOptions(java.lang.String[] options)
                throws java.lang.Exception
```

:   Parses a given list of options.


    Valid options are:

    ```
     -M <num>
      minimum allowable standard deviation for normal density computation 
      (default 1e-6)
    ```

    ```
     -W <clusterer name>
      Clusterer to wrap.
      (default weka.clusterers.SimpleKMeans)
    ```

    ```
     Options specific to clusterer weka.clusterers.SimpleKMeans:
    ```

    ```
     -N <num>
      number of clusters.
      (default 2).
    ```

    ```
     -V
      Display std. deviations for centroids.
    ```

    ```
     -M
      Replace missing values with mean/mode.
    ```

    ```
     -S <num>
      Random number seed.
      (default 10)
    ```

    :   **Specified by:**: `setOptions` in interface `OptionHandler`
    :   **Parameters:**: `options` - the list of options as an array of strings **Throws:**: `java.lang.Exception` - if an option is not supported

---


### getOptions

```
public java.lang.String[] getOptions()
```

:   Gets the current settings of the clusterer.

    :   **Specified by:**: `getOptions` in interface `OptionHandler`
    :   **Returns:**: an array of strings suitable for passing to setOptions()

---


### getRevision

```
public java.lang.String getRevision()
```

:   Returns the revision string.

    :   **Specified by:**: `getRevision` in interface `RevisionHandler` **Overrides:**: `getRevision` in class `AbstractClusterer`
    :   **Returns:**: the revision

---


### main

```
public static void main(java.lang.String[] argv)
```

:   Main method for testing this class.

    :   **Parameters:**: `argv` - the options


---


|  |  |  |
| --- | --- | --- |
| **Overview** \| **Package** \| **Class** \| **Tree** \| **Deprecated** \| **Index** \| **Help** \| **Weka's home** | |  |
| **PREV CLASS**   **NEXT CLASS** | **FRAMES**    **NO FRAMES**     **All Classes** |  |
| SUMMARY: NESTED \| FIELD \| CONSTR \| METHOD | DETAIL: FIELD \| CONSTR \| METHOD |  |


---
